# Dietary Exposure to Essential and Toxic Trace Elements in the Portuguese Population: A Total Diet Study Approach

**DOI:** 10.3390/foods15050838

**Published:** 2026-03-03

**Authors:** Marta Ventura, Andreia Rego, Sandra Gueifão, Inês Delgado, Inês Coelho

**Affiliations:** 1 Department of Food and Nutrition, National Institute of Health, Doutor Ricardo Jorge, Avenida Padre Cruz, 1649-016 Lisboa, Portugal; marta.ventura@insa.min-saude.pt (M.V.); andreia.rego@insa.min-saude.pt (A.R.); sandra.gueifao@insa.min-saude.pt (S.G.); ines.delgado@insa.min-saude.pt (I.D.); 2 MARE—Marine and Environmental Sciences Centre, ARNET—Aquatic Research Network Associate Laboratory, NOVA School of Science and Technology, NOVA University Lisbon, 2829-516 Caparica, Portugal; 3 Department of Chemical Engineering (DEQ), Instituto Superior Técnico, Avenida Rovisco Pais, 1049-001 Lisboa, Portugal

**Keywords:** diet, food groups, inorganic contaminants, micronutrients, Portugal

## Abstract

The World Health Organization (WHO) and the European Food Safety Authority (EFSA) recognise Total Diet Studies (TDS) as an essential public health tool for assessing dietary exposure to beneficial and harmful substances through food. This study provides occurrence data for nine trace elements (As, Cd, Co, I, Mo, Pb, Se, Sn, and Sr) in representative foods consumed by the Portuguese population, using a harmonised TDS methodology. The study also fills previously missing data in the Portuguese Food Composition Database, strengthening its value for nutritional monitoring and exposure assessment. The results show that the lowest concentration of all trace elements were observed in the “Water and water-based beverages” group while the highest concentrations were found in “Fish, seafood, amphibians, reptiles, and invertebrates” (As, Cd, I, Pb, Se, Sr), “Sugar confectionery and water-based sweet desserts” (Co), “Legumes, nuts, oilseeds and spices” (Mo), and “Fruit and fruit products” (Sn). Importantly, all measured levels of trace elements were below the applicable legal limits, indicating that the analysed foods do not pose a risk for consumer health. Furthermore, the data can support risk assessment, regulatory decisions, and the development of public health policies related to trace element intake. These findings also facilitate comparisons with international TDS data, contributing to global understanding of dietary exposures.

## 1. Introduction

Food, the primary source of nutrients, is essential for human survival and a key determinant of health. However, foods are complex matrices composed of many chemical substances. While they provide essential nutrients, foods can also contain contaminants harmful to human health, which can enter the food chain at different stages. Thus, it is crucial to properly characterise food components, generating data to estimate their contribution to daily allowances or risk assessment analysis, depending on whether they are nutrients or contaminants.

Total diet studies (TDS) are recognised by the World Health Organization (WHO) and the European Food Safety Authority (EFSA) as essential public health tools for assessing dietary exposure to beneficial and harmful substances through food [1]. These studies are based on food consumption survey data and involve the selection, collection (at retail level), and analysis of the most commonly consumed foods in a given country, representative of national dietary patterns. The TDS data are fundamental for estimating dietary exposure by combining occurrence data with food consumption data [1].

The present study is part of the first harmonised Portuguese TDS and provides a scientific basis to strengthen the link between food consumption and food composition in Portugal [1]. Micronutrients, such as selenium (Se), metals including cadmium (Cd) and lead (Pb), and metalloids such as arsenic (As), are priority substances among the chemical exposures addressed by the TDS methodology [1].

The TDS approach selected provides a realistic estimate of population dietary exposure based on the foods as consumed, while minimizing recall bias inherent to food frequency questionnaires. Compared with duplicate diet studies, TDS are more cost-effective and feasible for large-scale monitoring. In contrast to biomonitoring, TDS allows the direct identification of dietary sources of exposure and provides valuable information for risk assessment [1].

From a human health perspective, trace elements in food can be divided into three main groups: essential elements, non-essential elements, and toxic elements.

Essential elements, such as cobalt (Co), iodine (I), and selenium (Se), although required in trace amounts, play crucial roles in vital biological functions. Co is an essential trace element for humans and other animals, serving as a key component of vitamin B12 (cobalamin); however, excessive intake may be harmful to human health [2]. Selenium is an essential trace element, primarily through its incorporation into selenoproteins that regulate thyroid hormone metabolism, protect against oxidative stress, and support immune function [3]. Se acts as a cofactor of iodothyronine deiodinase, and its deficiency may impair the conversion of thyroxine (T4) to triiodothyronine (T3) [4]. I is an essential micronutrient for the synthesis and function of thyroid hormones and for the development of several organs. In particular, brain I deficiency is the most common cause of preventable intellectual disability worldwide, and there is a high risk of deficiency during pregnancy and infancy [5].

Non-essential elements, such as molybdenum (Mo) and strontium (Sr), are chemical elements not required for normal physiological function. Mo is a transition metal that acts as a cofactor for several important enzymes involved in important biochemical reactions [6]. Sr is a trace element with no recognized biological functions in humans; however, due to its chemical similarity to calcium, it can be absorbed by living organisms [7].

Toxic elements, such as As, Cd, Pb, and inorganic tin (Sn_i_), can occur naturally or be introduced through human activities throughout the Earth’s crust. This can cause environmental contamination and human exposure. These elements (As, Cd, Pb, and Sni) are toxic at the lowest concentrations and accumulate in human tissues [8,9,10]. As, Cd, and Pb are among the top ten chemicals or groups of chemicals of major public health concern as defined by the Agency for Toxic Substances and Disease Registry (ATSDR) [11]. Additionally, these toxic elements are classified as human carcinogens by the International Agency for Research on Cancer (IARC) [12].

This study aims to present results from the first harmonised Portuguese TDS, providing occurrence data for trace elements (As, Cd, Co, I, Mo, Pb, Se, Sn, and Sr) in representative foods consumed by the Portuguese population. By adopting a harmonised TDS methodology, this study aligns with international guidelines and enhances its global scientific relevance. Furthermore, it aims to contribute with previously missing data to the Portuguese Food Composition Database (FCD), and to generate an extensive dataset to be reported to EFSA, supporting the monitoring and updating of scientific information on dietary exposure. Importantly, the data generated by this study provide robust scientific evidence to support informed decision-making by public health policymakers, contributing to the development, evaluation, and refinement of food safety and nutrition policies. In the future, combined with national consumption data, the data obtained in this study will help estimate exposure to the analysed trace elements in the Portuguese population.

## 2. Materials and Methods

### 2.1. Sample Collection and Preparation

The sampling strategy was established according to the TDS methodology, considering the foods most consumed by the Portuguese population, as previously described in detail by Vasco et al. (2021) [13]. Food items were purchased and collected at the retail level from 2014 to 2016 in the Greater Lisbon region. Although the sampling period is restricted to 2014–2016, the reported data remain scientifically relevant, as these occurrence data are being published for the first time. Furthermore, this study contributes to the evaluation of the dietary exposure in the Portuguese population, particularly since no other TDS has been conducted in Portugal since 2015. To ensure the representativeness of TDS samples, each sample was composed of equal amounts (100 g) of 12 food items [13]. In short, 12 food items were purchased, prepared as consumed (edible part only, cooked or raw), and pooled into representative TDS samples.

The TDS samples were categorized into 17 food groups according to their nature, based on the FOODEX2 classification, namely: “Alcoholic beverages”; “Coffee, cocoa, tea and infusions”; “Composite dishes”; “Eggs and egg products”; “Fish, seafood, amphibians, reptiles and invertebrates”; “Fruit and fruit products”; “Fruit and vegetable juices and nectars”; “Grains and grain-based products”; “Legumes, nuts, oilseeds and spices”; “Meat and meat products”; “Milk and dairy products”; “Products for non-standard diets, food imitates and food supplements”; “Seasoning, sauces and condiments”; “Starchy roots or tubers and products thereof, sugar plants”; “Sugar confectionery and water-based sweet desserts”; “Vegetables and vegetable products”; and “Water and water-based beverages” [14].

The sampling plan included a total of 1956 food items collected and prepared as 163 pooled samples for laboratory analyses, and stored in a freezer at <−20 °C until analysis. After analysis, the 163 pooled samples were organized and grouped according to FoodEx2 subgroups; consequently, the number of samples per subgroup ranged from one to 26. This grouping approach allowed for systematic evaluation of trace element occurrence across the different dietary categories and facilitated comparison with international dietary exposure data.

### 2.2. Instrumentation

The laboratory analysis was performed using an ICP-MS (Inductively Coupled Plasma Mass Spectrometry, Thermo X series II, Thermo Fisher Scientific, Waltham, MA, USA) combined with an autosampler CETAC ASX-520 (CETAC Technologies, Omaha, Nebraska, USA). Each TDS sample was analysed for 9 trace elements using the following isotopes: ^75^As, ^111^Cd, ^59^Co, ^127^I, ^95^Mo, ^208^Pb, ^77^Se, ^88^Sr, and ^118^Sn.

The performance of the ICP-MS was evaluated and optimized daily using a tuning solution of 10 μg L^−1^ (Analytika, Prague, Czech Republic).

### 2.3. Reagents and Standards

All reagents used in this study were of ultrapure or suprapure grade. The ultrapure water used was obtained from a Milli-Q Element system (Millipore Corporation, Saint-Quentin, France). Pro analysis nitric acid (HNO_3_ 65%) was purchased and purified in the laboratory to ultrapure grade using an acid distillation system from Milestone SubPUR, ensuring its purity.

The calibration curves and internal quality control (QC) solutions for all trace elements, except I, were prepared using high-purity ICP-MS 1000 mg L^−1^ mono and multi-element stock standard solutions (Merck, Darmstadt, Germany). Standard solutions of germanium (Ge) containing 1000 mg L^−1^ (Inorganics Ventures, Christiansburg, VA, USA), yttrium (Y), and indium (In) containing 1000 mg L^−1^ (Merck) were used to prepare internal standards. Suprapur hydrogen peroxide (H_2_O_2_ 30%) was purchased from Merck. All dilutions and solutions were prepared using HNO_3_ 2% (*v*/*v*).

The calibration curve for I was prepared from a high-purity monoelementar stock solution containing 1000 mg L^−1^ (Inorganic Ventures). Rhodium (Rh) 10 mg L^−1^ and tellurium (Te) 1000 mg L^−1^ were purchased from Inorganics Ventures as internal standards.

Tetramethylammonium hydroxide 25% (*v*/*v*) (TMAH) was obtained from Fluka, Bucharest Romania. All solutions (calibration curve, internal quality control, blank solutions) and dilution of samples from the alkaline extraction were prepared with TMAH 0.5% (*v*/*v*).

Pancreatin from porcine pancreas was obtained from Sigma Aldrich, Darmstadt, Germany. A solution with Triton^®^ from Merck, Darmstadt, Germany, and ammonium hydroxide 30% from Avantor Performance Materials, was used to wash the ICP-MS sample introduction system after dilution to 0.1% (*v*/*v*) and addition of Triton 0.05% (*v*/*v*).

### 2.4. Acid Digestion

The analysis of As, Cd, Co, Mo, Pb, Se, Sr, and Sn was previously described by Coelho et al. (2013) [15]. In summary, approximately 0.5 g of solid samples or 2 g of liquid samples were weighed and combined with a mixture of 4 mL of concentrated ultrapure HNO_3_, 1 mL of suprapure H_2_O_2_, and 3 mL of ultrapure water in a Teflon closed-vessel. The vessels were then subjected to a microwave digestion process (Milestone Ethos 1 Series). The microwave program was as follows: (Step 1) 25 min to 90 °C, hold for 5 min; (Step 2) 15 min to 180 °C, hold for 10 min; (Step 3) 5 min to 210 °C, hold for 12 min; and (Step 4) 5 min to 90 °C, hold for 6 min. After digestion, and once the vessels had cooled to room temperature, the samples were transferred to centrifuge tubes, and the volume was adjusted to 25 mL with ultrapure water.

### 2.5. Alkaline Extraction

For I determination, the methodology followed the procedure described by Delgado et al. (2019) [16]. An overview of this method shows that the alkaline extraction consisted of several steps before analysis: (Step 1) weigh around 0.5 g of each solid TDS sample or 2 g of each liquid TDS sample into a 50 mL centrifuge plastic tube, (Step 2) add 1 mL of TMAH 25% (*v*/*v*) and 8 mL of ultrapure water for solid samples or 1 mL of TMAH 25% (*v*/*v*) for liquid samples, (Step 3) place tubes in a hot graphite block system (DigiPREP, SCP Science) for 3 h at 90 °C, (Step 4) centrifuge at 10,000 rpm for 10 min and (Step 5) filter through 0.45 µm syringe filters.

For samples with an expected high starch content, a 2% (*v*/*v*) pancreatin solution at 37 °C was added and left overnight before extraction. After extraction, all samples were diluted with ultrapure water to a final volume of 25 mL.

### 2.6. Statistical Analysis

Each pool was analysed in triplicate, and the result was calculated as the mean value ± standard deviation as presented in Supplementary Table S1.

Following analysis, the pools were organised into 17 food groups and 62 subgroups according to the FoodEx2 classification system, as previously mentioned. Within each subgroup, the results are summarised as minimum, maximum, and mean values, providing a clear overview of trace element occurrence across the different dietary categories.

### 2.7. Quality Assurance

The analytical procedures were conducted under quality assurance conditions, following the guidelines outlined in EN ISO/IEC 17025:2017 [17].

Sample quantification was performed using a calibration curve with at least five concentration points, covering the previously validated working ranges for each element. Each analytical sequence included the analysis of instrumental blanks and QC samples at the start and end of the sequence, ensuring robust monitoring of drift and contamination throughout the analysis. Additionally, a digestion blank was included in each batch of samples to detect and account for any potential contamination introduced during sample preparation.

Table 1 summarizes the figures of merit for the analytical method attained under rigorous quality control conditions. The working ranges established for each element align well with the characteristics of the study matrices, ensuring precise and accurate quantification within the specified limits. Repeatability, expressed as the relative standard deviation (RSD), consistently remained below 10% across all elements.

To ensure method accuracy, fortified samples, spiked with a known amount of certified standards for all analysed trace elements, were included in each digestion cycle. Recovery rates for spiked samples ranged globally from 80% to 120%, per element, for the analysed food groups, in accordance with the laboratory’s quality control scheme. Measurement uncertainties were calculated annually using analytical internal quality control data across the various food matrices, demonstrating robust, reliable performance.

Throughout the study, the laboratory participated regularly in Proficiency Testing Schemes for similar food matrices, except for Sr, for which these programmes were unavailable. Satisfactory z-scores were observed for all elements.

## 3. Results and Discussion

Table 2 presents the results of As, Co, I, Mo, Se, and Sr in foods consumed by the Portuguese population according to the FoodEx2 classification at the food subgroup level.

Trace elements such as Cd, Pb, and Sn were not included in Table 2, as Cd and Pb were below the limit of quantification (LOQ) in 85% of the analysed samples, and Sn was below the LOQ in 70% of the samples. Nevertheless, Cd and Pb reached their highest concentrations in the “Fish, seafood, amphibians, reptiles, and invertebrates” group, with quantifiable levels observed in 28% and 16% of samples, respectively. Sn presented its highest concentrations in the “Fruit and fruit products” group and showed elevated levels in the “Fine bakery wares” subgroup of the “Grains and grain-based products” group, as well as quantifiable concentrations in 56% of the “Dishes, incl. Ready-to-eat meals” group. Importantly, all measured concentrations of Cd, Pb, and Sn were below the applicable legal limits for the respective food groups, highlighting that dietary exposure to these elements does not pose a risk to consumers [18].

Individual concentrations of As, Cd, Co, I, Mo, Pb, Se, Sn, and Sr in each analysed TDS sample are presented in the Supplementary Table S1.

The results will first be discussed per trace element, by comparing the mean values across food subgroups and with results reported in the literature, followed by an evaluation of the individual samples. It should be noted that comparisons with published studies are indicative, as external factors —such as culinary treatments, geographical origin (soil and climate), agricultural practices, and climate change—may influence the chemical composition of the analysed samples.

### 3.1. Arsenic

Of the 162 samples analysed, As was quantifiable in 52% of cases. The lowest mean concentration of As was observed in the soft drinks subgroup (1.8 µg kg^−1^), whereas the highest mean concentration was observed in the molluscs subgroup (4450 µg kg^−1^).

The results for the “Alcoholic beverages” group are in consistent with international TDS data [19], although they are lower than those reported in the Danish FCD [20].

The “Grains and grain-based products” group, with the exception of the rice grains subgroup, aligns with the findings from other TDS studies [19], while the bread and similar products subgroup exhibited higher As levels than those reported in the Danish FCD [20].

For the coffee beverages in the “Coffee, cocoa, tea, and infusions” group, the mean value obtained (3.6 µg kg^−1^) was below those reported by Millour et al. (2011) [19] but in line with the Danish FCD [20].

The food groups “Seasoning, sauces and condiments”, “Sugar confectionery and water-based sweet desserts”, and “Water and water-based beverages” presented values lower than those reported in the literature [19].

Within the “Fish, seafood, amphibians, reptiles and invertebrates” group, marine fish was the most contaminated subgroup with the maximum level of As reaching 13,964 ± 930 µg kg^−1^ (conger, European species) (Supplementary Table S1) followed by the molluscs subgroup with a maximum concentration of 9138 ± 119 µg kg^−1^ (octopus, common) (Supplementary Table S1), and Shrimps and prawns (mean 4104 ± 160 µg kg^−1^). Compared to the literature, the mean values of the fish subgroups (diadromous fish, freshwater fish, and marine fish) are similar to other TDS [19]. However, when compared with the Danish FCD, the concentrations observed in Atlantic salmon, catfish, European plaice, ling, and marine shrimps or prawns were lower, whereas other demersal marine fishes, octopus, and hakes exhibited higher concentrations than those reported in the literature. The following samples were consistent with the the Danish FCD: Nile perch, horse mackerel, fresh tuna, and canned fish in oil, as shown in Supplementary Table S1 [20]. The predominant chemical form of As in fish and seafood is the organic form, arsenobetaine [21,22], which is a non-toxic form and does not pose a risk to human health; however, the high levels of As in European Conger point to an aquatic environmental impact. Conger is a fish species considered an indicator of marine pollution biomonitorization due to its sedentary behaviour and high trophic level [23]. The present result suggests a potential contamination of the aquatic environment. The differences between the results of this study and those reported in the literature for the ‘Fish, seafood, amphibians, reptiles, and invertebrates’ group could be attributed species-specific and trophic differences, as well as differences in habitat and environmental variability.

The levels of As present in fish were also evident in other food groups, particularly within the “composite dishes” group. High As concentrations were observed, especially in the “dishes, including ready-to-eat meals” subgroup, with a maximum concentration of 7869 ± 160 µg kg^−1^ observed in the Fish and potatoes meal (mixed fish stew) (Supplementary Table S1). The elevated As concentrations in this subgroup were mainly associated with dishes prepared with fish, such as this sample, which contained conger.

The As levels observed in rice grains and in fruit juices and nectars were below the legal limits for inorganic As established by the European Union (2023) [18]. However, the As levels in rice grains were higher (62 µg kg^−1^) than that reported by Millour et al. (2011) [19] but lower than the values reported in the EFSA report (2021) [24].

These As data form the basis of the first harmonised TDS dietary exposure assessment conducted in Portugal [25].

### 3.2. Cobalt

Co presented quantifiable levels in 30% of the 154 food samples analysed. The lowest concentration of Co was observed in drinking water (1.0 µg kg^−1^), which is in agreement with the literature [26], followed by meat stock cubes or granulates (1.6 µg kg^−1^), which were below values reported in the literature. The highest concentration was observed in chocolate, and chocolate products (122 µg kg^−1^), which aligns with other TDS studies [26].

In the “Alcoholic beverages” group, wine showed concentrations consistent with the literature [27], whereas beer exhibited levels below those previously reported [26]. The coffee beverages in the “Coffee, cocoa, tea, and infusions” group were consistent with the literature; however, tea infusions presented lower concentrations than those reported previously [26].

A similar pattern was observed in the “Fish, seafood, amphibians, reptiles, and invertebrates” group: the “Shrimps and prawns” subgroup aligned with the literature [20,26], whereas the “Molluscs” subgroup exhibited concentrations higher than those reported in other TDS studies [26], but consistent with the Danish FCD [20].

Within the “Fruit and fruit products” group, jam exhibited higher Co concentration than those reported in the literature [26]. In the “Grains and grain-based products “group, the processed and mixed breakfast cereals subgroup deserves particular attention, as it showed concentrations exceeding those reported in comparable TDS studies [26]. “Table-top condiments”, as well as the “Flowering brassica”, “Brassica vegetables”, and “Pulses (dry seeds)” subgroups, were consistent with literature values [26].

Although no tolerable upper intake level (UL) has been established for Co, the concentrations measured in this study—including the highest levels observed in chocolate and chocolate products—indicate that the contribution of individual food items to overall dietary Co exposure is unlikely to pose a risk to consumers.

### 3.3. Iodine

Of the 139 food samples analysed, I was quantifiable in 71%. The highest I concentrations were observed in the “Fish, seafood, amphibians, reptiles, and invertebrates” group, particularly in the “Shrimps and prawns” (714 µg kg^−1^) and “Molluscs” (642 µg kg^−1^) subgroups. Hen eggs (243 µg kg^−1^) and dairy products, especially firm-ripened cheeses (394 µg kg^−1^), also presented notable I concentrations.

Shrimps and prawns exhibited I concentrations higher than those reported in the Danish FCD (76–128 µg kg^−1^) but substantially lower than values reported in the French FCD (2600 µg kg^−1^). In contrast, molluscs showed lower mean I concentrations compared with the French and Danish FCD (1000 µg kg^−1^ and 2190 µg kg^−1^, respectively), as well as values reported by Chung et al. (2013) (970 µg kg^−1^) [20,28,29]. These discrepancies between the present study and the literature for the “Fish, seafood, amphibians, reptiles, and invertebrates” group may be attributed to differences in species composition and geographical origin. The I content determined in hen eggs was consistent with the range reported by the Danish FCD (230–295 µg kg^−1^) but lower than values reported in other studies, which ranged from 325 to 530 µg kg^−1^ [20,27,28,30]. Among dairy products, spoonable dairy desserts exhibited relevant I concentrations (227 µg kg^−1^), closely aligning with values reported by Cressey (2003) [31]. The mean I content of firm-ripened cheeses (394 µg kg^−1^) was higher than values reported for Edam cheese in the Czech FCD (60–70 µg kg^−1^), but comparable to ranges reported for other cheese varieties in the Danish FCD (104.4–180 µg kg^−1^) and by Johannesen et al. (2023) [20,32,33].

The lowest quantified I concentration was observed in still natural mineral water (2.5 µg kg^−1^). Additionally, the food groups “Vegetables and vegetable products”, “Fruits and fruit products”, “Legumes, nuts, oilseeds, and spices” generally exhibited I concentrations below the LOQ, with only a few exceptions.

The recommended daily intake (RDI) of I ranges between 70 to 130 µg day^−1^ [5]. The results for the “Shrimps and prawns” and “Molluscs” subgroups contributed the highest proportion to the RDI of I, reaching up to 50%. These findings are consistent with previous studies, which commonly identify seafood, dairy products, and eggs as the main contributors to I intake, whereas vegetables, fruits, and beverages generally contain lower I concentrations [30,34,35]. Furthermore, the UL for I established by the EFSA is 600 µg day^−1^ for adults [36]. The results obtained in this study do not indicate a risk of excessive I intake for consumers.

### 3.4. Molybdenum

Mo was quantified in 66% of the 155 food samples analysed. The lowest concentration of Mo was observed in still natural mineral water (1.6 µg kg^−1^). The highest concentration was observed in peanuts (1865 µg kg^−1^), which is above the results reported in the literature [26]. The “Alcoholic beverages” group, most of the subgroups of the “Composite dishes” group, and the “Eggs and egg products” group showed values consistent with the literature.

In the “Coffee, cocoa, tea, and infusions” group, particular attention should be given to the coffee beverages subgroup, which showed levels of Mo above the literature [26], whereas the “Cocoa ingredients” subgroup presented values below literature reports [20]. The “Dishes, incl. ready-to-eat meals” subgroup presented values in line with the literature. In contrast, the levels of Mo observed in the “Pulses (dry seeds)” subgroup were higher than those reported in the literature [20].

The “Molluscs” and “Soft drinks” subgroups presented results lower than those reported in the literature; on the other hand, the “Potato-boiled” subgroup aligns with the results reported in the literature, as well as most of the samples in the “Vegetables and vegetable products” group [26]. The Mo concentrations observed in the “Grains and grain-based products” group, as well as the “Sausages and other comminuted meat” subgroup, and all subgroups from the “Milk and dairy products” group, presented values in line with the results reported in the literature [20,26].

The UL for Mo established by the EFSA is 600 µg day^−1^ for adults [36]. Considering the concentrations measured in this study, even food items with elevated Mo levels, such as peanuts, are unlikely to exceed the UL under typical dietary consumption patterns. Nevertheless, the higher Mo concentrations observed in certain food subgroups, particularly “Pulses (dry seeds)” and “Coffee beverages”, may contribute substantially to total dietary Mo intake in high consumers [6]. This highlights the importance of continued monitoring to ensure that long-term exposure remains below the established UL.

### 3.5. Selenium

Se was present in 74% of the analysed samples (*n* = 163). The lowest Se concentration was observed in drinking water (0.58 µg kg^−1^), which was lower than values reported in the literature [26]. The RDI for Se is 70 µg/day [3]. Samples from the “Fish, seafood, amphibians, reptiles, and invertebrates” group were the main contributors to the RDI of Se. The results for this food group ranged from 141 ± 3 µg kg^−1^ (terrestrial snails, edible) to 694 ± 39 µg kg^−1^ (shrimps and prawns) (Supplementary Table S1); the latter exceeded values published in FCDs [20,29,37].

The values of Se in the “Marine fish” subgroup ranged from 376 ± 16 µg kg^−1^ (cod Atlantic) to 1057 ± 23 µg kg^−1^ (tuna) (Supplementary Table S1), and are mostly consistent with literature values [29] except for the “Other coastal marine fish” subgroup, which exhibited higher concentrations than reported [20,29].

The “Meat and meat products” group is the second largest contributor to the RDI of Se, with results generally aligning with the literature [29]. However, Se concentration in rabbit fresh meat samples (312 ± 9 µg kg^−1^) were lower than reported in the literature, whereas Se concentrations in raw cured meat (774 ± 70 µg kg^−1^), dried and fermented sausages (335 ± 0 µg kg^−1^), and Frankfurter-type sausages (275 ± 3 µg kg^−1^) were higher [29].

Hen eggs also presented high Se concentration (339 µg kg^−1^), exceeding values reported in the Italian and Danish FCDs [20,37].

However, the UL for Se established by the EFSA is 255 µg day^−1^ for adults [4]. The concentrations observed in this study do indicate a risk of Se overexposure.

### 3.6. Strontium

Sr was present in all 144 food samples analysed in this study. The lowest Sr concentration was observed in drinking water (6.7 µg kg^−1^), which is below values reported in the literature [38,39]. The highest concentration was observed in terrestrial snails (13,519 µg kg^−1^), followed by shrimps and prawns (12,258 µg kg^−1^).

The “Freshwater fish” and “Marine fish” subgroups (778 µg kg^−1^ and 1992 µg kg^−1^, respectively) are consistent with values reported in the literature for fish (1100 µg kg^−1^) [39]. In contrast, the “Molluscs” subgroup (6927 µg kg^−1^) presents values below those reported in the literature (10,350 µg kg^−1^) [39]. The lower Sr concentrations observed in molluscs may reflect differences in species composition, habitat characteristics, or environmental Sr availability.

Compared to the literature, elevated Sr concentrations were also found in subgroups such as “Dried fruit”, “Pulses”, “Firm cheeses”, and “Leafy vegetables” [39]. Higher Sr levels in dried fruits, pulses, and leafy vegetables may be associated with soil geochemistry and agricultural practices. Strontium uptake by plants is influenced by soil Sr content, calcium availability, and fertilization practices. Differences in soil composition and irrigation water may therefore explain the elevated concentrations compared with previous studies [7].

## 4. Conclusions

The results of the present study integrate the first harmonized Portuguese TDS. Even though the sampling period is a limitation, as it took place between 2014 and 2016, the data reported are still relevant to date, since these are being published for the first time, contributing to the assessment of dietary exposure of the Portuguese population, and no other TDS has been conducted in Portugal since 2015. This study reinforces the importance of conducting periodic national TDS.

The food group with the lowest concentrations of the analysed trace elements (As, Cd, Co, I, Mo, Pb, Se, Sn, and Sr) was the “Water and water-based beverages” group. On the other hand, the food groups with the highest levels of the analysed trace elements were: “Fish, seafood, amphibians, reptiles, and invertebrates” for As, Cd, I, Pb, Se, and Sr, “Sugar confectionery and water-based sweet desserts” for Co, “Legumes, nuts, oilseeds and spices” for Mo, and “Fruit and fruit products” for Sn.

When combined with national consumption data, the results obtained in this study contribute to estimating the dietary exposure of the Portuguese population to the analysed trace elements. The data also contributes to updating the Portuguese FCD by adding new parameters to existing food items and incorporating new food items.

The results of this study provide a more accurate and comprehensive assessment of dietary exposure to trace elements, which is essential for public health monitoring and risk assessment. Moreover, this occurrence data may provide a scientific basis for policy formulation, enabling regulatory authorities to establish or refine maximum permissible levels for trace elements in foods, design control strategies, and prioritize monitoring programs.

## Figures and Tables

**Table 1:** Analytical figures of merit for multielemental determination by ICP-MS.

Element	LOD (µg L^−1^)	LOQ (µg L^−1^)	Working Range (µg L^−1^)	Repeatability (RSD, %)	Recovery (%)	Uncertainty ^(1)^ (%)	PT Schemes (z-Score)
As	0.01	0.10	0.25–2.5	≤8.3	89–119	18–28	−0.1
Cd	0.01	0.10	0.25–2.5	≤5.6	93–117	14–22	0.3
Co	0.02	0.10	0.25–2.5	≤5.4	87–107	15–21	0.2
I	0.15	0.47	0.5–50	≤8.4	89–111	17–23	−0.1
Mo	0.10	0.34	0.5–5.0	≤7.0	104–120	14–27	0.2
Pb	0.02	0.25	0.5–5.0	≤3.2	93–114	21–28	−0.2
Se	0.02	0.40	0.5–5.0	≤7.8	84–107	15–27	0.2
Sn	0.04	0.13	0.25–2.5	≤10	82–119	14–23	0.2
Sr	0.03	0.25	0.5–5.0	≤7.6	95–117	10–25	n.a.

n.a. Not available; PT proficiency testing schemes; ^(1)^ Expanded uncertainty (coverage factor of 2).

**Table 2:** Levels of As, Co, I, Mo, Se, and Sr in foods consumed by the Portuguese population (values in µg kg^−1^ fresh weight).

Food Group and Subgroup	As				Co				I				Mo				Se				Sr			
	n	Min.	Max.	Mean	n	Min.	Max.	Mean	n	Min.	Max.	Mean	n	Min.	Max.	Mean	n	Min.	Max.	Mean	n	Min.	Max.	Mean
**Alcoholic beverages**																								
Beer	1	-	-	5.0	1	-	-	<1.6 *	1	-	-	15	1	-	-	14	1	-	-	<3.1 *	1	-	-	83
Wine	1	-	-	2.4	1	-	-	4.8	0	-	-	-	1	-	-	5.9	1	-	-	<3.1 *	1	-	-	674
**Coffee, cocoa, tea and infusions**																								
Cocoa ingredients	1	-	-	11	1	-	-	62	0	-	-	-	1	-	-	48	1	-	-	11	1	-	-	944
Coffee beverages	1	-	-	3.6	1	-	-	10	0	-	-	-	1	-	-	46	1	-	-	<3.1 *	1	-	-	133
Herbal and other non-tea infusions	1	-	-	<1.6 *	1	-	-	<1.6 *	0	-	-	-	1	-	-	<3.1 *	1	-	-	<3.1 *	1	-	-	180
Tea infusion (black, white)	1	-	-	<1.6 *	1	-	-	1.8	0	-	-	-	1	-	-	<3.1 *	1	-	-	<3.1 *	1	-	-	99
**Composite dishes**																								
Dishes, incl. ready to eat meals	26	<24 *	7869	417	22	<25 *	26	<25 *	21	<32 *	282	91	26	<24 *	510	119	26	26	186	100	24	479	2644	1535
Salads	2	<13 *	49	26	2	<13 *	13	<13 *	2	<12 *	40	24	2	<26 *	74	45	2	<26 *	87	53	2	618	1927	1273
Soups (ready-to-eat)	6	<13 *	259	47	3	<13 *	19	<13 *	6	<12 *	150	40	6	<26 *	144	45	6	<26 *	47	34	3	623	3674	1755
**Eggs and egg products**																								
Hen eggs	1	-	-	<13 *	1	-	-	<13 *	1	-	-	243	1	-	-	101	1	-	-	339	1	-	-	279
**Fish, seafood, amphibians, reptiles and invertebrates**																								
Diadromous fish	1	-	-	680	1	-	-	<35 *	1	-	-	123	1	-	-	<70 *	1	-	-	430	1	-	-	920
Fish and seafood (processed)	4	761	2069	1206	4	<35 *	<35 *	<35 *	4	197	565	321	4	<70 *	85	<70 *	4	217	688	473	4	1143	8523	4331
Freshwater fish	2	46	70	58	2	<35 *	<35 *	<35 *	2	28	131	80	2	<70 *	<70 *	<70 *	2	242	251	246	2	714	843	778
Marine fish	13	809	13,964	2847	13	<35 *	<35 *	<35 *	13	97	1378	403	13	<70 *	<70 *	<70 *	13	376	1057	692	12	963	3467	1992
Molluscs	3	543	9138	4450	3	<18 *	289	112	3	131	1569	642	3	<36 *	140	64	3	301	612	459	3	3696	13,365	6927
Shrimps and prawns	1	-	-	4104	1	-	-	40	1	-	-	714	1	-	-	<36 *	1	-	-	694	1	-	-	12,258
Terrestrial snails, edible	1	-	-	29	1	-	-	63	1	-	-	59	1	-	-	182	1	-	-	141	1	-	-	13,519
**Fruit and fruit products**																								
Dried fruit	2	21	31	26	2	<12 *	46	<12 *	2	<17 *	55	34	2	77	93	85	2	<24 *	<24 *	<24 *	2	2632	5958	4295
Fresh fruit	9	<12 *	<12 *	<12 *	9	<12 *	17	<12 *	9	<38 *	<38 *	<38 *	9	<24 *	76	<24 *	9	<24 *	<24 *	<24 *	9	196	1872	777
Jam	1	-	-	<12 *	1	-	-	12	1	-	-	<17 *	1	-	-	<24 *	1	-	-	<24 *	1	-	-	852
Other processed fruit products (excluding beverages)	2	<12 *	<12 *	<12 *	2	<12 *	12	<12 *	2	<36 *	268	131	2	<24 *	<24 *	<24 *	2	<24 *	<24 *	<24 *	2	696	992	844
**Fruit and vegetable juices and nectars**																								
Fruit juices and nectars	2	<2.0 *	3.8	2.5	2	2.9	20	11	2	5.6	7.5	6.5	2	7.4	15	11	2	<4.0 *	<4.0 *	<4.0 *	2	453	614	533
**Grains and grain-based products**																								
Bread and similar products	3	<12 *	16	13	1	-	-	<25 *	3	<12 *	37	16	3	139	213	180	3	54	75	62	1	-	-	1124
Fine bakery wares	14	<12 *	24	15	14	<25 *	98	<25 *	10	42	288	101	14	76	149	111	14	35	134	66	9	688	2553	1228
Pasta and similar products	1	-	-	<12 *	1	-	-	<25 *	1	-	-	7.5	1	-	-	112	1	-	-	80	0	-	-	-
Popcorn (maize, popped)	1	-	-	<12 *	1	-	-	<25 *	0	-	-	-	1	-	-	123	1	-	-	37	1	-	-	1036
Processed and mixed breakfast cereals	1	-	-	26	1	-	-	48	1	-	-	8.5	1	-	-	209	1	-	-	52	0	-	-	-
Rice grains (p)	1	-	-	62	1	-	-	<25 *	1	-	-	<7.7 *	1	-	-	191	1	-	-	<50 *	1	-	-	632
**Legumes, nuts, oilseeds and spices**																								
Legumes fresh seeds	2	<24 *	<24 *	<24 *	2	29	68	49	2	<8.6 *	<8.6 *	<8.6 *	2	394	942	668	2	<48 *	<48 *	<48 *	2	1253	2287	1770
Peanut	1	-	-	<9.1 *	1	-	-	74	0	-	-	-	1	-	-	1865	1	-	-	96	0	-	-	-
Pulses (dry seeds)	4	<24 *	43	<24 *	4	<24 *	41	28	4	<2.3 *	3.3	<2.3 *	3	208	720	536	4	91	196	144	4	2985	7930	4641
Table olives ready for consumption	1	-	-	<24 *	1	-	-	<24 *	1	-	-	41	1	-	-	<48 *	1	-	-	251	1	-	-	8316
**Meat and meat products**																								
Generic non-game mammals fresh meat	5	<24 *	<24 *	<24 *	5	<24 *	<24 *	<24 *	5	<20 *	78	37	5	<48 *	<48 *	<48 *	5	116	312	187	5	659	869	753
Poultry fresh meat	2	<24 *	30	<24 *	2	<24 *	<24 *	<24 *	2	<20 *	24	<20 *	2	<48 *	67	<48 *	2	285	375	330	2	419	648	533
Processed whole meat products	2	<24 *	<24 *	<24 *	2	<24 *	<24 *	<24 *	1	-	-	33	2	<48 *	<48 *	<48 *	2	142	774	458	2	406	948	677
Sausages and other comminuted meat	2	<24 *	30	<24 *	2	<24 *	<24 *	<24 *	2	133	134	134	2	<48 *	70	53	2	275	335	305	2	1437	1566	1502
**Milk and dairy products**																								
Dairy desserts (spoonable)	1	-	-	<10 *	1	-	-	<10 *	1	-	-	227	1	-	-	70	1	-	-	76	1	-	-	360
Fermented milk or cream	2	<8.6 *	<8.6 *	<8.6 *	2	<8.6 *	<8.6 *	<8.6 *	2	172	179	175	2	39	41	40	2	18	24	21	0	-	-	-
Firm—ripened cheeses	1	-	-	<25 *	1	-	-	<25 *	1	-	-	394	1	-	-	64	1	-	-	157	1	-	-	3756
Milk	2	<8.6 *	8.9	<8.6 *	2	<8.6 *	17	<8.6 *	2	158	199	178	2	35	40	37	2	18	39	28	1	-	-	327
**Products for non-standard diets, food imitators and food supplements**																								
Meat imitates	1	-	-	53	1	-	-	26	1	-	-	<20 *	1	-	-	717	1	-	-	147	1	-	-	1851
Soya drink	1	-	-	8.1	1	-	-	12	1	-	-	5.9	1	-	-	197	1	-	-	14	1	-	-	829
**Seasoning, sauces and condiments**																								
Stock cubes or granulate, meat	1	-	-	45	1	-	-	1.6	1	-	-	23	1	-	-	28	1	-	-	86	1	-	-	55
Table-top condiments	4	<6.0 *	21	11	4	<6.0	12	7.6	3	15	47	26	0	-	-	-	4	<12 *	90	39	4	214	1891	917
**Starchy roots or tubers and products thereof, sugar plants**																								
Potato boiled	1	-	-	<12 *	1	-	-	<12 *	1	-	-	<3.4 *	1	-	-	32	1	-	-	<25 *	1	-	-	399
**Sugar confectionery and water-based sweet desserts**																								
Chocolate and chocolate products	1	-	-	11	1	-	-	122	0	-	-	-	0	-	-	-	1	-	-	110	1	-	-	2694
Gelatine dessert	1	-	-	2.2	1	-	-	<2.1 *	1	-	-	18	1	-	-	<4.2 *	1	-	-	6.4	1	-	-	79
White sugar	1	-	-	<12 *	1	-	-	<12 *	0	-	-	-	0	-	-	-	1	-	-	<24 *	1	-	-	87
**Vegetables and vegetable products**																								
Asparagus	1	-	-	<11 *	1	-	-	17	1	-	-	65	1	-	-	28	1	-	-	31	1	-	-	647
Beans, green with pods	1	-	-	<11 *	1	-	-	15	1	-	-	<17 *	1	-	-	216	1	-	-	42	1	-	-	1428
Brassica vegetables	3	<13 *	13	<13 *	3	6.4	8.5	7.2	3	<17 *	<17 *	<17 *	3	41	154	115	3	<25 *	28	<25 *	3	1151	4338	2344
Carrots	1	-	-	<11 *	1	-	-	<11 *	1	-	-	<17 *	1	-	-	22	1	-	-	24	1	-	-	2585
Common/Portobello/Champignon mushroom	1	-	-	78	1	-	-	<8.5 *	1	-	-	<16 *	1	-	-	29	1	-	-	208	1	-	-	520
Cucurbits fruiting vegetables	3	<8.5 *	<8.5 *	<8.5 *	3	<8.5 *	12	<8.5 *	3	<3.1 *	<3.1 *	<3.1 *	3	<17 *	36	24	3	<17 *	<17	<17 *	3	133	627	342
Flowering brassica	2	<13 *	<13 *	<13 *	2	13	19	16	2	<23 *	<23 *	<23 *	2	34	45	39	2	<25 *	<25 *	<25 *	2	719	1976	1347
Leafy vegetables	3	<10 *	<10 *	<10 *	3	<10 *	12	<10 *	3	<17 *	22	<17 *	3	<20 *	89	59	3	21	32	26	3	2153	8981	6380
Onion bulb	1	-	-	<11 *	1	-	-	<11 *	1	-	-	<17 *	1	-	-	<23 *	1	-	-	<23 *	1	-	-	1139
Solanacea fruiting vegetables	2	<11 *	12	<11 *	2	<11 *	23	15	2	<3.1 *	3.7	<3.1 *	2	50	53	51	2	<22 *	<22 *	<22 *	2	452	647	549
Sweet Corn canned	0	-	-	-	1	-	-	<9.0 *	1	-	-	<16 *	0	-	-	-	1	-	-	82	1	-	-	199
**Water and water-based beverages**																								
Drinking water	1	-	-	<0.25 *	1	-	-	1.0	0	-	-	-	1	-	-	<0.50 *	1	-	-	0.58	1	-	-	6.7
Soft drinks	3	<1.6 *	4.0	1.8	3	<1.6 *	<1.6 *	<1.6 *	0	-	-	-	3	<3.1 *	18	7.2	3	<3.1 *	4.0	<3.1 *	3	26	74	43
Still natural mineral water	1	-	-	2.9	1	-	-	<0.25 *	1	-	-	2.5	1	-	-	1.6	1	-	-	0.63	1	-	-	17

*—limit of quantification.

## Data Availability

The original contributions presented in this study are included in the article/Supplementary Materials. Further inquiries can be directed to the corresponding author.

## Supplementary Materials

The following supporting information can be downloaded at: https://www.mdpi.com/article/10.3390/foods15050838/s1, Supplementary Table S1: The levels of trace elements (As, Cd, Co, I, Mo, Pb, Se, Sn, and Sr) in Portuguese TDS samples, expressed in fresh weight as the mean ± standard deviation in µg kg^−1^.
