# Supplementary material for: Dietary Exposure to Essential and Toxic Trace Elements in the Portuguese Population: A Total Diet Study Approach
Source: Foods. 2026 Mar 3;15(5):838. doi: 10.3390/foods15050838 (PMC12984638; doi:10.3390/foods15050838)
Supplement: Supplementary file 1 [file foods-15-00838-s001.zip › foods-4080400-supplementary.pdf]

Supplementary material

Assessment of trace elements in the Portuguese Diet: A Total Diet Study Approach

Marta Ventura[a,b], Andreia Rego[a,c], Sandra Gueifão[a], Inês Delgado[a], Inês Coelho[a,*]

[a]Department of Food and Nutrition, National Institute of Health Doutor Ricardo Jorge, INSA. IP, Avenida Padre Cruz, 1649-016 Lisboa, Portugal

[b]MARE - Marine and Environmental Sciences Centre, ARNET - Aquatic Research Network Associate Laboratory, and Department of Applied Social Sciences, NOVA School of Science and Technology, NOVA University Lisbon, Caparica, Portugal

[c]Department of Chemical Engineering (DEQ), Instituto Superior Técnico, Avenida Rovisco Pais, 1049-001 Lisboa, Portugal

*Corresponding author, e-mail address: ines.coelho@insa.min-saude.pt (Inês Coelho)

Table S1. The levels of trace elements (As, Cd, Co, I, Mo, Pb, Se, Sn, and Sr) in Portuguese TDS samples, expressed in fresh weight as the mean ± standard deviation in μg kg^{-1}.

Food group	Food subgroup	TDS name	As	Cd	Co	I	Mo	Pb	Se	Sn	Sr
			**x** ± SD (μg kg^{-1})								
Alcoholic beverages	Beer	Beer	5.0 ± 0.1	<1.6*	<1.6*	15.1 ± 0.3	14.4 ± 0.2	<3.1*	<3.1*	<1.6*	83 ± 2
Alcoholic beverages	Wine	Wine	2.40 ± 0.03	<1.6*	4.8 ± 0.2	n.a.	5.9 ± 0.3	22 ± 1	<3.1*	<1.6*	674 ± 6
Coffee, cocoa, tea and infusions	Cocoa ingredients	Cocoa ingredients	11.2 ± 0.2	16 ± 2	62 ± 4	n.a.	48 ± 4	11.5 ± 0.4	11.1 ± 0.2	2.5 ± 0.3	944 ± 62
Coffee, cocoa, tea and infusions	Coffee beverages	Coffee beverages	3.6 ± 0.2	<1.6*	10.4 ± 0.2	n.a.	46 ± 3	7 ± 1	<3.1*	<1.6*	133 ± 4
Coffee, cocoa, tea and infusions	Herbal and other non-tea infusions	Herbal and other non-tea infusions	<1.6*	<1.6*	<1.6*	n.a.	<3.1*	<3.1*	<3.1*	<1.6*	180 ± 2
Coffee, cocoa, tea and infusions	Tea infusion (black, white)	Tea infusion (black, white)	<1.6*	2.9 ± 0.1	1.81 ± 0.04	n.a.	<3.1*	9 ± 1	<3.1*	<1.6*	99 ± 9
Composite dishes	Dishes, incl. Ready to eat meals (excluding soups and salads)	Dishes, incl. Ready to eat meals (excluding soups and salads) (bread based fish)	123 ± 2	<13*	<13*	282 ± 56	52 ± 1	<25*	84 ± 3	<13*	2347 ± 110
Composite dishes	Dishes, incl. Ready to eat meals (excluding soups and salads)	Potato based dishes (mashed potatoes)	<12*	13 ± 0.3	<25*	56 ± 11	45 ± 1	<25*	26 ± 1	<13*	479 ± 23

Food group	Food subgroup	TDS name	As	Cd	Co	I	Mo	Pb	Se	Sn	Sr
			x ± SD (µg kg^{-1})								
Composite dishes	Dishes, incl. Ready to eat meals (excluding soups and salads)	Potatoes and meat meal (portato puree with meat)	<12*	<13*	n.a.	21.8 ± 0.3	55 ± 1	<25*	64.9 ± 0.2	38 ± 3	n.a.
Composite dishes	Dishes, incl. Ready to eat meals (excluding soups and salads)	Beans and meat meal (beans and gut dish)	<12*	<13*	24.7 ± 0.1	29 ± 6	510 ± 6	<25*	62 ± 4	719 ± 10	1900 ± 75
Composite dishes	Dishes, incl. Ready to eat meals (excluding soups and salads)	Beans, meat, and vegetables meal (meat and vegetables boiled dishes, Portuguese style)	15.9 ± 0.4	<13*	<12*	32 ± 6	95 ± 3	<25*	95 ± 6	11 ± 8	1501 ± 70
Composite dishes	Dishes, incl. Ready to eat meals (excluding soups and salads)	Beans, meat, and vegetables meal (beans stewed with pork and cow meat)	<12*	<13*	13.2 ± 0.1	n.a.	236 ± 3	<25*	60 ± 6	467 ± 21	1621 ± 104
Composite dishes	Dishes, incl. Ready to eat meals (excluding soups and salads)	Beans, meat, and vegetables meal (pork mest, chickpea, pasta and vegetables stewed)	<12*	<13*	16.0 ± 0.3	18 ± 4	327 ± 11	<25*	90 ± 8	408 ± 40	2438 ± 146

Food group	Food subgroup	TDS name	As	Cd	Co	I	Mo	Pb	Se	Sn	Sr
			x ± SD (μg kg^{-1})								
Composite dishes	Dishes, incl. Ready to eat meals (excluding soups and salads)	Meat based dishes	24.6 ± 0.1	<13*	<12*	n.a.	79 ± 2	<25*	97 ± 9	214 ± 20	1086 ± 50
Composite dishes	Dishes, incl. Ready to eat meals (excluding soups and salads)	Meat burger (no sandwich)	<24*	<24*	<24*	34.6 ± 0.3	55 ± 2	<48*	114 ± 10	15 ± 2	1003 ± 46
Composite dishes	Dishes, incl. Ready to eat meals (excluding soups and salads)	Meat balls	<24*	<24*	<24*	83 ± 6	155 ± 4	<48*	88 ± 6	503 ± 11	1347 ± 17
Composite dishes	Dishes, incl. Ready to eat meals (excluding soups and salads)	Fish and seafood based dishes	189 ± 2	<13*	<12*	159 ± 13	119 ± 2	<25*	62 ± 4	<13*	2075 ± 75
Composite dishes	Dishes, incl. Ready to eat meals (excluding soups and salads)	Seafood-based meals	419 ± 7	27 ± 1	26 ± 1	103 ± 3	80 ± 2	<25*	76 ± 4	79 ± 5	2644 ± 110
Composite dishes	Dishes, incl. Ready to eat meals (excluding soups and salads)	Prepared fish salad	216 ± 11	<13*	<12*	84± 1	140 ± 3	<25*	136 ± 8	<13*	2169 ± 28

Food group	Food subgroup	TDS name	As	Cd	Co	I	Mo	Pb	Se	Sn	Sr
			x ± SD (μg kg^{-1})								
Composite dishes	Dishes, incl. Ready to eat meals (excluding soups and salads)	Fish and potatoes meal (cod fish dishes)	97 ± 2	<13*	15.4 ± 0.9	236 ± 6	78 ± 3	<25*	113 ± 6	12 ± 4	2105 ± 109
Composite dishes	Dishes, incl. Ready to eat meals (excluding soups and salads)	Fish and potatoes meal (mixed fish stew)	7869 ± 160	13.19 ± 0.04	12 ± 2	129 ± 26	<24*	<25*	156 ± 6	15 ± 1	1729 ± 24
Composite dishes	Dishes, incl. Ready to eat meals (excluding soups and salads)	Fish and potatoes meal (potato puree with fish)	175 ± 10	<13*	n.a.	92.0 ± 0.1	52 ± 1	<25*	113 ± 10	620 ± 24	1279 ± 79
Composite dishes	Dishes, incl. Ready to eat meals (excluding soups and salads)	Fish and rice meal	1301 ± 11	16.6 ± 0.1	n.a.	84 ± 8	58 ± 1	<25*	74 ± 6	649 ± 28	1641 ± 52
Composite dishes	Dishes, incl. Ready to eat meals (excluding soups and salads)	Quiche	<13*	<13*	<13*	n.a.	81 ± 2	<26*	145 ± 3	<13*	1127 ± 29
Composite dishes	Dishes, incl. Ready to eat meals (excluding soups and salads)	Omelette, plain	<12*	<13*	<12*	180 ± 14	75 ± 2	<25*	153 ± 8	<13*	888 ± 9

Food group	Food subgroup	TDS name	As	Cd	Co	I	Mo	Pb	Se	Sn	Sr
			x ± SD (µg kg^{-1})								
Composite dishes	Dishes, incl. Ready to eat meals (excluding soups and salads)	Sandwich with meat and vegetable topping/filling	12.8 ± 0.8	<13*	n.a.	99 ± 20	62 ± 1	<25*	114 ± 11	55 ± 2	1555 ± 44
Composite dishes	Dishes, incl. Ready to eat meals (excluding soups and salads)	Pizza and pizza-like dishes	15 ± 1	<13*	20 ± 2	63 ± 13	130 ± 2	<25*	120 ± 25	847 ± 7	2134 ± 16
Composite dishes	Dishes, incl. Ready to eat meals (excluding soups and salads)	Finger food	35 ± 3	<13*	<14*	n.a.	165 ± 10	<28*	186 ± 16	<13*	n.a.
Composite dishes	Dishes, incl. Ready to eat meals (excluding soups and salads)	Lasagna	16.4 ± 0.8	<13*	<12*	n.a.	87 ± 2	<25*	80 ± 6	<13*	1193 ± 75
Composite dishes	Dishes, incl. Ready to eat meals (excluding soups and salads)	Rice based dishes cooked	171 ± 9	<13*	<13*	71 ± 14	95 ± 1	<26*	146.4 ± 0.3	1054 ± 33	1109 ± 37
Composite dishes	Dishes, incl. Ready to eat meals (excluding soups and salads)	Rice and vegetables meal	36 ± 2	<13*	<12*	13 ± 1	158 ± 4	<24*	40 ± 4	n.a.	788 ± 26

Food group	Food subgroup	TDS name	As	Cd	Co	I	Mo	Pb	Se	Sn	Sr
			x ± SD (μg kg^{-1})								
Composite dishes	Dishes, incl. Ready to eat meals (excluding soups and salads)	Rice and meat meal	46 ± 0.71	<13*	<13*	43 ± 9	90 ± 1	<26*	97 ± 9	<13*	678 ± 9
Composite dishes	Salads	Mixed vegetable salad (tomato and lettuce salad)	<12*	<13*	13 ± 6	9 ± 1	<24*	<24*	<24*	n.a.	618 ± 56
Composite dishes	Salads	Mixed vegetable salad (Russian salad)	49 ± 1	<13*	<13*	40 ± 8	74 ± 1	<26*	87 ± 2	<13*	1927 ± 43
Composite dishes	Soups (ready-to-eat)	Tomato soup	<12*	<13*	n.a.	32 ± 6	33 ± 1	<24*	35 ± 3	<13*	n.a.
Composite dishes	Soups (ready-to-eat)	Legume (beans) soup	<12*	<13*	n.a.	6.9 ± 0.4	144 ± 12	<24*	34 ± 1	<13*	n.a.
Composite dishes	Soups (ready-to-eat)	Mixed vegetables soup, green cabbage soup	<12*	<13*	19 ± 2	14 ± 3	<24*	<24*	28 ± 2	n.a.	968 ± 60
Composite dishes	Soups (ready-to-eat)	Mixed vegetables soup, with puree or pieces	<12*	<13*	n.a.	6.62 ± 0.03	25.3 ± 0.5	<24*	<24*	<13*	n.a.
Composite dishes	Soups (ready-to-eat)	Meat soup, with pieces (chicken soup)	<13*	<13*	<13*	29 ± 6	32.7 ± 0.3	<26*	47 ± 1	<13*	623 ± 22
Composite dishes	Soups (ready-to-eat)	Fish soup	259 ± 8	<13*	<12*	150 ± 10	<24*	<25*	44 ± 3	357 ± 22	3674 ± 138
Eggs and egg products	Hen eggs	Hen eggs	<13*	<13*	<13*	243 ± 22	101.4 ± 0.5	<26*	339 ± 13	<13*	279 ± 7

Food group	Food subgroup	TDS name	As	Cd	Co	I	Mo	Pb	Se	Sn	Sr
			x ± SD (µg kg^{-1})								
Fish, seafood, amphibians, reptiles and invertebrates	Diadromous fish	Salmon, Atlantic	680 ± 32	<36*	<35*	123 ± 5	<70*	<70*	430 ± 15	<36*	920 ± 58
Fish, seafood, amphibians, reptiles and invertebrates	Fish and seafood processed	Cod, dried	1060 ± 16	<36*	<35*	565 ± 10	<70*	<71*	491 ± 8	<36*	6373 ± 86
Fish, seafood, amphibians, reptiles and invertebrates	Fish and seafood processed	Fish fingers, breaded	934 ± 40	<17*	<17*	281 ± 2	85 ± 2	<34*	217 ± 4	<17*	1283 ± 43
Fish, seafood, amphibians, reptiles and invertebrates	Fish and seafood processed	Canned fish in oil (tuna)	761 ± 12	<36*	<35*	197 ± 6	<70*	<71*	688 ± 28	<36*	1143 ± 21
Fish, seafood, amphibians, reptiles and invertebrates	Fish and seafood processed	Canned fish in oil (sardines)	2069 ± 69	<36*	<35*	243 ± 1	<70*	<71*	497 ± 26	119 ± 10.8	8523 ± 350
Fish, seafood, amphibians, reptiles and invertebrates	Freshwater fish	Catfishes (freshwater)	70 ± 3	<36*	<35*	28 ± 3	<70*	<71*	242 ± 9	<36*	843 ± 45
Fish, seafood, amphibians, reptiles and invertebrates	Freshwater fish	Perch, Nile	46 ± 3	<36*	<35*	131 ± 3	<70*	<71*	251 ± 6	<36*	714 ± 21

Food group	Food subgroup	TDS name	As	Cd	Co	I	Mo	Pb	Se	Sn	Sr
			x ± SD (μg kg^{-1})								
Fish, seafood, amphibians, reptiles and invertebrates	Freshwater fish	Conger, European	13964 ± 930	<36*	<35*	225 ± 7	<70*	<70*	816 ± 15	<36*	963 ± 22
Fish, seafood, amphibians, reptiles and invertebrates	Marine fish	Sea bream	850 ± 27	<36*	<35*	173 ± 4	<70*	<70*	663 ± 42	<36*	1045 ± 48
Fish, seafood, amphibians, reptiles and invertebrates	Marine fish	Other coastal marine fishes (Wrasse, Pouting, Black Spot, Sea Bream, Red Porgy)	2340 ± 44	<36*	<35*	1310 ± 14	<70*	<70*	807 ± 49	<36*	3337 ± 498
Fish, seafood, amphibians, reptiles and invertebrates	Marine fish	Plaice, European	2722 ± 44	<36*	<35*	193 ± 2	<70*	<71*	481 ± 11	<36*	3467 ± 149
Fish, seafood, amphibians, reptiles and invertebrates	Marine fish	Cod, Atlantic	3544 ± 58	<22*	12 ± 11.93	1378 ± 6	<45*	<45*	376 ± 16	<36*	2784 ± 2
Fish, seafood, amphibians, reptiles and invertebrates	Marine fish	Hakes	1728 ± 50	<36*	<35*	97 ± 2	<70*	<71*	413 ± 4	101 ± 4	1653 ± 116
Fish, seafood, amphibians, reptiles and invertebrates	Marine fish	Ling	1451 ± 48	<36*	<35*	272 ± 13	<70*	<71*	646 ± 6	<36*	1492 ± 9

Food group	Food subgroup	TDS name	As	Cd	Co	I	Mo	Pb	Se	Sn	Sr
			x ± SD (μg kg^{-1})								
Fish, seafood, amphibians, reptiles and invertebrates	Marine fish	Horse mackerel	1783 ± 35	<36*	<35*	444 ± 10	<70*	<71*	630 ± 46	<36*	n.a.
Fish, seafood, amphibians, reptiles and invertebrates	Marine fish	Mackerel, chub	1261 ± 25	<22*	<22*	307 ± 9	<45*	<45*	902 ± 36	<22*	2334 ± 163
Fish, seafood, amphibians, reptiles and invertebrates	Marine fish	Sardine, European	2638 ± 79	25 ± 1	<22*	214 ± 6	<45*	<45*	852 ± 51	<22*	3325 ± 100
Fish, seafood, amphibians, reptiles and invertebrates	Marine fish	Tuna	809 ± 18	<36*	<35*	167 ± 3	<70*	<71*	1057 ± 23	<36*	1097 ± 24
Fish, seafood, amphibians, reptiles and invertebrates	Marine fish	Other demersal marine fishes (forkbeard, redfish)	2651 ± 64	<22*	<22*	215 ± 4	<45*	<45*	623 ± 7	<22*	1222 ± 85
Fish, seafood, amphibians, reptiles and invertebrates	Marine fish	Other pelagic marine fishes (scabbardfish)	1269 ± 14	<36*	<35*	249 ± 19	<70*	<71*	730 ± 26	<36*	1180 ± 36
Fish, seafood, amphibians, reptiles and invertebrates	Molluscs	Bivalve molluscs	3667 ± 234	125 ± 1	289 ± 9	1569 ± 58	140 ± 1	260 ± 26	612 ± 4	32 ± 1	13,365 ± 889

Food group	Food subgroup	TDS name	As	Cd	Co	I	Mo	Pb	Se	Sn	Sr
			**x** ± SD (μg kg^{-1})								
Fish, seafood, amphibians, reptiles and invertebrates	Molluscs	Octopus, common	9138 ± 119	115 ± 3	30 ± 2	131 ± 5	<36*	<36*	301 ± 19	<18*	3696 ± 124
Fish, seafood, amphibians, reptiles and invertebrates	Molluscs	Squid, common	543 ± 13	507 ± 36	17 ± 6	225 ± 5	43 ± 1	<36*	464 ± 43	<18*	3720 ± 109
Fish, seafood, amphibians, reptiles and invertebrates	Shrimps and prawns	Marine shrimps or prawns, cooked	4104 ± 160	93.5 ± 0.3	40 ± 1	714 ± 22	<36*	<36*	694 ± 39	<18*	12,258 ± 375
Fish, seafood, amphibians, reptiles and invertebrates	Terrestrial snails, edible	Terrestrial snails, edible	29 ± 1	248 ± 3	63 ± 3	59 ± 1	182 ± 1	282 ± 6	141 ± 3	<18*	13,519 ± 943
Fruit and fruit products	Dried fruit	Dried vine fruits (raisins etc.)	21 ± 2	<12*	<12*	55 ± 4	77 ± 1	<24*	<24*	<12*	2632 ± 177
Fruit and fruit products	Dried fruit	Dried figs	31 ± 2	<12*	46 ± 2	<17*	93 ± 6	<24*	<24*	<12*	5958 ± 298
Fruit and fruit products	Fresh fruit	Orange, sweet	<12*	<12*	<12*	<3.1*	<24*	<24*	<24*	<12*	1095 ± 55
Fruit and fruit products	Fresh fruit	Apple	<12*	<12*	<12*	<17*	<24*	<24*	<24*	<12*	246 ± 7
Fruit and fruit products	Fresh fruit	Pear	<12*	<12*	13 ± 1	<17*	<24*	<24*	<24*	<12*	196 ± 10
Fruit and fruit products	Fresh fruit	Table-grapes	<12*	<12*	<12*	<17*	<24*	<24*	<24*	<12*	980 ± 59

Food group	Food subgroup	TDS name	As	Cd	Co	I	Mo	Pb	Se	Sn	Sr
			**x** ± SD (μg kg^{-1})								
Fruit and fruit products	Fresh fruit	Strawberry	<12*	<12*	<12*	<38*	76 ± 7	<25*	<24*	<12*	585 ± 19
Fruit and fruit products	Fresh fruit	Peach	<12*	<12*	<12*	<37*	<24*	<24*	<24*	4674 ± 71	422 ± 1
Fruit and fruit products	Fresh fruit	Kiwifruit	<12*	<12*	17 ± 8	<37*	<24*	<24*	<24*	<12*	1872 ± 107
Fruit and fruit products	Fresh fruit	Banana	<12*	<12*	<12*	<38*	<24*	<24*	<24*	<12*	623 ± 33
Fruit and fruit products	Fresh fruit	Pineapple	<12*	<12*	12.0 ± 0.7	<37*	<24*	<24*	<24*	<12*	972 ± 82
Fruit and fruit products	Jam	Jam	<12*	<12*	12.5 ± 0.5	<17*	<24*	<24*	<24*	<12*	852 ± 9
Fruit and fruit products	Other processed fruit products (excluding beverages)	Fruit Salad	<12*	<12*	<12*	268 ± 11	<24*	<24*	<24*	1493 ± 27	696 ± 42
Fruit and fruit products	Other processed fruit products (excluding beverages)	Canned or jarred fruit	<12*	<12*	12.0 ± 0.7	<36*	<24*	<24*	<24*	72200 ± 463	992 ± 14
Fruit and vegetable juices and nectars (including concentrates)	Fruit juices and nectars	Fruit juices	<2*	<2*	19.9 ± 0.5	7 ± 1	15 ± 1	<4*	<4*	<2*	614 ± 5

Food group	Food subgroup	TDS name	As	Cd	Co	I	Mo	Pb	Se	Sn	Sr
			**x** ± SD (µg kg^{-1})								
Fruit and vegetable juices and nectars (including concentrates)	Fruit juices and nectars	Fruit nectars	3.75 ± 0.08	<2*	2.93 ± 0.06	5.6 ± 0.1	7.4 ± 0.2	<4*	<4*	<2*	453 ± 1
Grains and grain-based products	Bread and similar products	Single grain bread and rolls	<12*	<12*	<24*	<7.8*	139 ± 9	25.3 ± 0.5	54 ± 3	<12*	n.a.
Grains and grain-based products	Bread and similar products	Wheat bread and rolls, white (refined flour)	13.6 ± 0.2	13.3 ± 0.2	n.a.	8.9 ± 0.4	189 ± 5	<25*	59 ± 5	<12*	n.a.
Grains and grain-based products	Bread and similar products	Crackers	16.2 ± 0.9	14 ± 0.2	n.a.	37 ± 7	213 ± 6	<25*	75 ± 7	<12*	1124 ± 97
Grains and grain-based products	Fine bakery wares	Biscuits, sweet, plain	<12*	<12*	<12*	n.a.	149 ± 4	<25*	48 ± 2	<12*	1049 ± 46
Grains and grain-based products	Fine bakery wares	Biscuits, chocolate	<12*	18 ± 1.09	98 ± 1	58 ± 12	129 ± 3	<25*	35 ± 3	<12*	2553 ± 163
Grains and grain-based products	Fine bakery wares	Cakes	20 ± 1	<12*	<12*	n.a.	97 ± 3	<26*	79 ± 7	<12*	1062 ± 35
Grains and grain-based products	Fine bakery wares	Sponge cake	22.2 ± 0.9	<12*	<25*	288 ± 58	140 ± 2	<25*	120 ± 7	<12*	n.a.
Grains and grain-based products	Fine bakery wares	Chocolate cakes	18.1 ± 1	<12*	71 ± 2	122 ± 24	100 ± 3	<24*	83 ± 1	84 ± 1	1320 ± 53

Food group	Food subgroup	TDS name	As	Cd	Co	I	Mo	Pb	Se	Sn	Sr
			x ± SD (µg kg^{-1})								
Grains and grain-based products	Fine bakery wares	Fruit cake	<12*	<12*	<12*	n.a.	98 ± 1	<25*	57 ± 5	4927 ± 33	961 ± 88
Grains and grain-based products	Fine bakery wares	Muffins	19.4 ± 0.1	<12*	<25*	107 ± 21	115 ± 4	<25*	54 ± 3	20 ± 1	n.a.
Grains and grain-based products	Fine bakery wares	Yeast leavened pastry (leavened cake)	23.7 ± 0.8	<12*	<12*	48 ± 1	117 ± 6	<25*	48 ± 5	<12*	688 ± 62
Grains and grain-based products	Fine bakery wares	Yeast leavened pastry (brioche)	22 ± 2	<12*	<12*	42 ± 2	133 ± 5	<25*	55 ± 2	<12*	1294 ± 74
Grains and grain-based products	Fine bakery wares	Croissant	23 ± 1	<12*	22.5 ± 0.8	47 ± 2	97 ± 2	<25*	62 ± 3	<12*	1064 ± 71
Grains and grain-based products	Fine bakery wares	Shortcrust (pies - tarts)	13.5 ± 0.9	<12*	<12*	n.a.	97 ± 2	<26*	134 ± 2	228 ± 23	1062 ± 35
Grains and grain-based products	Fine bakery wares	Flan tart	<9*	<12*	<9*	131 ± 26	76 ± 1	<18*	58 ± 6	10 ± 2	n.a.
Grains and grain-based products	Fine bakery wares	Fruit pie-tarts	<12*	<12*	20.8 ± 0.6	81 ± 16	85 ± 1	<25*	45 ± 2	5281 ± 210	n.a.
Grains and grain-based products	Fine bakery wares	Various pastry	<12*	<12*	<25*	81 ± 16	123 ± 9	<25*	52.0 ± 0.0	<12*	n.a.
Grains and grain-based products	Pasta and similar products	Pasta and similar products	<12*	<12*	<25*	8 ± 1	112 ± 5	<25*	80 ± 4	<12*	n.a.

Food group	Food subgroup	TDS name	As	Cd	Co	I	Mo	Pb	Se	Sn	Sr
			**x** ± SD (µg kg^{-1})								
Grains and grain-based products	Popcorn (maize, popped)	Popcorn (maize, popped)	<12*	<12*	<12*	n.a.	123 ± 12	<25*	37 ± 7	<12*	1036 ± 10
Grains and grain-based products	Processed and mixed breakfast cereals	Processed and mixed breakfast cereals	26 ± 3	13.8 ± 0.4	48 ± 1	9 ± 1	209 ± 4	<25*	52 ± 2	n.a.	n.a.
Grains and grain-based products	Rice grains (p)	Rice grains (p)	62 ± 4	<24*	<25*	<7.7*	191 ± 1	<50*	<50*	<24*	632 ± 28
Legumes, nuts, oilseeds and spices	Legumes fresh seeds	Broad bean (fresh seeds)	<24*	<24*	68 ± 6	<8.6*	942 ± 23	<48*	<50*	<24*	1253 ± 54
Legumes, nuts, oilseeds and spices	Legumes fresh seeds	Peas (fresh seeds, without pods)	<24*	<24*	29 ± 1	<2.3*	394 ± 5	<48*	<50*	<24*	2287 ± 105
Legumes, nuts, oilseeds and spices	Peanut	Peanut	<9*	60 ± 5	74 ± 3	n.a.	1865 ± 65	<18*	96 ± 5	<9*	n.a.
Legumes, nuts, oilseeds and spices	Pulses (dry seeds)	Beans (dry seeds)	<24*	<24*	41 ± 1	<2.3*	720 ± 15	<48*	91 ± 8	38 ± 2	2985 ± 42
Legumes, nuts, oilseeds and spices	Pulses (dry seeds)	Chickpea (dry seeds)	20.5 ± 0.2	<12*	30.2 ± 0.4	<2.2*	n.a.	<22*	112 ± 5	27 ± 1	4437 ± 184
Legumes, nuts, oilseeds and spices	Pulses (dry seeds)	Cowpea (dry seeds)	<24*	<24*	<24*	3.3 ± 0.5	680 ± 15	<48*	176 ± 9	17 ± 1	3210 ± 13
Legumes, nuts, oilseeds and spices	Pulses (dry seeds)	Lupin (dry seeds)	43 ± 3	<24*	<24*	2.7 ± 0.1	208 ± 3	<48*	196 ± 1	18.2 ± 0.5	7930 ± 87

Food group	Food subgroup	TDS name	As	Cd	Co	I	Mo	Pb	Se	Sn	Sr
			**x** ± SD (μg kg^{-1})								
Legumes, nuts, oilseeds and spices	Table olives ready for consumption	Table olives for consumption	<24*	<24*	<24*	41 ± 1	<48*	51 ± 4	251 ± 20	62 ± 2	8316 ± 294
Meat and meat products	Generic non-game mammals fresh meat	Bovine fresh meat	<24*	<24*	<24*	<20*	<48*	<48*	142 ± 9	<24*	729 ± 16
Meat and meat products	Generic non-game mammals fresh meat	Calf fresh meat	<24*	<24*	<24*	<20*	<48*	<48*	116 ± 10	<24*	659 ± 3
Meat and meat products	Generic non-game mammals fresh meat	Swine fresh meat	<24*	<24*	<24*	<20*	<48*	<48*	217 ± 7	<24*	734 ± 34
Meat and meat products	Generic non-game mammals fresh meat	Sheep fresh meat	<24*	<24*	<24*	75 ± 4	<48*	<45*	148 ± 7	<24*	775 ± 58
Meat and meat products	Generic non-game mammals fresh meat	Rabbit fresh meat	<24*	<24*	<24*	78 ± 4	<48*	<48*	312 ± 9	220 ± 4	869 ± 110
Meat and meat products	Poultry fresh meat	Chicken fresh meat	<24*	<24*	<24*	24 ± 2	67 ± 1	<48*	375 ± 37	<24*	648 ± 63
Meat and meat products	Poultry fresh meat	Turkey fresh meat	30 ± 3	<24*	<24*	<20*	<48*	<45*	285 ± 4	<24*	419 ± 4
Meat and meat products	Processed whole meat products	Raw cured meat (ham and bacon)	<12*	<12*	n.a.	n.a.	<24*	<24*	774 ± 70	<12*	948 ± 59
Meat and meat products	Processed whole meat products	Cooked cured meat (cooked ham)	<24*	<24*	<24*	33 ± 1	<48*	<45*	142 ± 5	<24*	406 ± 33

Food group	Food subgroup	TDS name	As	Cd	Co	I	Mo	Pb	Se	Sn	Sr
			**x** ± SD (µg kg^{-1})								
Meat and meat products	Sausages and other comminuted meat	Dry and fermented sausages	30 ± 1	<24*	<24*	134 ± 11	<48*	38 ± 43	335 ± 0	<24*	1566 ± 66
Meat and meat products	Sausages and other comminuted meat	Frankfurter type sausage	<24*	<24*	<24*	133 ± 9	70 ± 2	<48*	275 ± 3	399 ± 9	1437 ± 218
Milk and dairy products	Dairy desserts spoonable	Dairy desserts spoonable	<10*	<10*	<10*	227.1 ± 0.3	70 ± 2	<20*	76 ± 7	27 ± 1	360 ± 4
Milk and dairy products	Fermented milk or cream	Yoghurt	<9*	<10*	<9*	179 ± 2	39 ± 2	<17*	18.4 ± 0.4	<10*	n.a.
Milk and dairy products	Fermented milk or cream	Acidophilus milk	<9*	<10*	<9*	172 ± 2	41 ± 1	<17*	24 ± 2	70 ± 1	n.a.
Milk and dairy products	Firm - ripened cheeses	Firm - ripened cheeses	<25*	<24*	<25*	394 ± 20	64 ± 2	<50*	157 ± 4	<24*	3756 ± 217
Milk and dairy products	Milk	Cow milk	8.9 ± 0.3	<3*	<3*	158 ± 12	40 ± 3	<6*	39 ± 1	n.a.	326.9 ± 0.1
Milk and dairy products	Milk	Flavoured milks	<9*	<10*	17 ± 1	199 ± 3	34.8 ± 0.3	<17*	18 ± 1	<10*	n.a.
Products for non-standard diets, food imitates and food supplements	Meat imitates	Meat imitates	53 ± 1	<10*	26.1 ± 0.4	<20*	717 ± 67	35 ± 1	147 ± 11	251 ± 13	1851 ± 127

Food group	Food subgroup	TDS name	As	Cd	Co	I	Mo	Pb	Se	Sn	Sr
			**x** ± SD (µg kg^{-1})								
Products for non-standard diets, food imitates and food supplements	Soya drink	Soya drink	8.1 ± 0.2	5.5 ± 0.2	11.6 ± 0.1	5.9 ± 0.2	197 ± 4	5.6 ± 0.5	14 ± 1	<1.6*	829 ± 27
Seasoning, sauces and condiments	Stock cubes or granulate, meat	Stock cubes or granulate, meat	45 ± 1	<1.6*	1.59 ± 0.02	23 ± 5	28 ± 3	<3*	86 ± 5	<1.6*	55 ± 3
Seasoning, sauces and condiments	Table-top condiments	Vinegar	4.5 ± 0.1	<3*	5.2 ± 0.2	15 ± 3	n.a.	<3*	<3.1*	3.0 ± 0.1	480 ± 5
Seasoning, sauces and condiments	Table-top condiments	Tomato ketchup	8.3 ± 0.4	<10*	11.4 ± 0.8	15 ± 3	n.a.	<10*	19 ± 2	<5*	1082 ± 34
Seasoning, sauces and condiments	Table-top condiments	Mayonnaise	11 ± 1	<12*	<6*	47 ± 1	n.a.	<12*	44 ± 3	8 ± 0.6	214 ± 4
Seasoning, sauces and condiments	Table-top condiments	Other common table-top condiments	21 ± 1	<10*	12.1 ± 0.6	n.a.	n.a.	20 ± 2	90 ± 6	16 ± 1	1891 ± 1
Starchy roots or tubers and products thereof, sugar plants	Potato boiled	Potato boiled	<12*	13.7 ± 0.3	<12*	<3.4*	31.5 ± 0.3	<25*	<25*	<12*	399 ± 12
Sugar, confectionery and water-based sweet desserts	Chocolate and chocolate products	Chocolate and chocolate products	11 ± 1	20.2 ± 0.5	122 ± 1	n.a.	n.a.	<13*	110 ± 1	<5*	2694 ± 30

Food group	Food subgroup	TDS name	As	Cd	Co	I	Mo	Pb	Se	Sn	Sr
			x ± SD (µg kg^{-1})								
Sugar, confectionery and water-based sweet desserts	Gelatine dessert	Gelatine dessert	2.2 ± 0.1	0 ± 0	<2*	17.6 ± 0.4	<4*	<4.2*	6 ± 1	<2.1*	79.0 ± 0.5
Sugar, confectionery and water-based sweet desserts	White sugar	White sugar	<12*	<24*	<12*	n.a.	n.a.	<24*	<25*	<12*	87 ± 3
Vegetables and vegetable products	Asparagus	Asparagus	<11*	<10*	17 ± 1	65 ± 4	27.6 ± 0.2	<23*	31 ± 1	25 ± 0.5	647 ± 6
Vegetables and vegetable products	Beans, green with pods	Beans, green with pods	<11*	<10*	14.6 ± 0.3	<17*	216 ± 4	<22*	42 ± 2	<10*	1428 ± 43
Vegetables and vegetable products	Brassic vegetables	Brussel sprouts	10.5 ± 0.2	<10*	<10*	<17*	151 ± 16	<20*	28 ± 2	<10*	1151 ± 45
Vegetables and vegetable products	Brassic vegetables	White cabbage	<13*	<10*	<13*	<17*	41 ± 1	<13*	<25*	n.a.	1542 ± 26
Vegetables and vegetable products	Brassic vegetables	Portuguese cabbage	13 ± 1	<10*	<10*	<17*	154 ± 13	23 ± 2	24 ± 2	<10*	4338 ± 431
Vegetables and vegetable products	Carrots	Carrot	15 ± 1	<10*	3 ± 0,79	<17*	22 ± 1	32 ± 2	24 ± 1	<10*	2585 ± 52
Vegetables and vegetable products	Common/Portobello/Champignon mushroom	Common/Portobello/Champignon mushroom	78 ± 2	<10*	<9*	<16*	29 ± 1	<17*	208 ± 3	37 ± 2	520 ± 7

Food group	Food subgroup	TDS name	As	Cd	Co	I	Mo	Pb	Se	Sn	Sr
			x ± SD (μg kg^{-1})								
Vegetables and vegetable products	Cucurbits fruiting vegetables	Melons (except watermelon)	<9*	<10*	<9*	<3.1*	36 ± 1	<17*	<17*	<10*	627 ± 25
Vegetables and vegetable products	Cucurbits fruiting vegetables	Common melon varieties	<9*	<10*	11.9 ± 0.2	<3.1*	<17*	<17*	<17*	<10*	266 ± 16
Vegetables and vegetable products	Cucurbits fruiting vegetables	Watermelons	<9*	<10*	<9*	<2.9*	21.9 ± 0.3	<17*	<17*	<10*	133 ± 3
Vegetables and vegetable products	Flowering brassica	Broccoli	<13*	<13*	19 ± 1	<23*	45 ± 3	<13*	<25*	n.a.	1976 ± 29
Vegetables and vegetable products	Flowering brassica	Cauliflower	<13*	<13*	13 ± 1	<23*	34 ± 1	<13*	<25*	n.a.	719 ± 14
Vegetables and vegetable products	Leafy vegetables	Lettuce	<10*	<10*	<10*	22.2 ± 0.4	<23*	<20*	21 ± 1	<10*	2153 ± 129
Vegetables and vegetable products	Leafy vegetables	Other leafy vegetables (Rapini)	<10*	11 ± 1	12 ± 0.2	<17*	70 ± 4	<20*	32 ± 1	<10*	8981 ± 359
Vegetables and vegetable products	Leafy vegetables	Turnip greens	<10*	12 ± 1	<10*	<17*	89 ± 4	<20*	24 ± 1	<10*	8006 ± 320
Vegetables and vegetable products	Onion bulb	Onions bulb	<11*	<10*	<11*	<17*	<23*	<23*	<23*	<10*	1139 ± 114
Vegetables and vegetable products	Solanacea fruiting vegetables	Tomato and similar (p)	<11*	<10*	<11*	<3.3*	50 ± 2	<23*	<23*	<10*	452 ± 18

Food group	Food subgroup	TDS name	As	Cd	Co	I	Mo	Pb	Se	Sn	Sr
			x ± SD (μg kg^{-1})								
Vegetables and vegetable products	Solanacea fruiting vegetables	Peppers, sweet	12 ± 1	<10*	22.5 ± 0.2	3.7 ± 0.3	53 ± 1	<22*	<23*	52 ± 3	647 ± 19
Vegetables and vegetable products	Sweet Corn canned	Sweet Corn canned	n.a.	<18*	<9*	<16*	n.a.	<18*	82 ± 1	50 ± 1.8	199 ± 1
Water and water-based beverages	Drinking water	Water cooking 4 G (Tap Water)	<0.25*	<0.25*	1.04 ± 0.01	n.a.	<0.5*	0.86 ± 0.02	0.6 ± 0.1	<0.25*	6.7 ± 0.2
Water and water-based beverages	Soft drinks	Soft drink, mixed flavours	<1.6*	<1.6*	<1.6*	n.a.	<3.1*	<3.1*	<3.1*	<1.6*	30.2 ± 0.1
Water and water-based beverages	Soft drinks	Soft drink, orange flavour	<1.6*	<1.6*	<1.6*	n.a.	18 ± 3	<3.1*	4.0 ± 0.4	<1.6*	74 ± 4
Water and water-based beverages	Soft drinks	Cola beverages, caffeinic	<1.6*	<1.6*	<1.6*	n.a.	3.1 ± 0.3	<3.1*	<3.1*	<1.6*	25.7 ± 0.2
Water and water-based beverages	Still natural mineral water	Still natural mineral water	2.9 ± 0.06	<0.25*	<0.25*	2.5 ± 0.3	1.55 ± 0.03	<0.5*	0.63 ± 0.06	<0.25*	17 ± 1

* - limit of quantification, n.a. - not available
